# Global gene expression analysis data of chicken dendritic cells infected with H9N2 avian influenza virus

**DOI:** 10.1016/j.dib.2020.105430

**Published:** 2020-03-16

**Authors:** Qingtao Liu, Jing Yang, Xinmei Huang, Yuzhuo Liu, Kaikai Han, Dongmin Zhao, Lijiao Zhang, Yin Li

**Affiliations:** Institute of Veterinary Medicine, Jiangsu Academy of Agricultural Sciences, and Key Laboratory of Veterinary Biological Engineering and Technology, Ministry of Agriculture, Nanjing, Jiangsu, China

**Keywords:** Global gene expression, Chicken, Dendritic cells, H9N2 avian influenza virus

## Abstract

This data article reports the global gene expression analysis data of chicken DCs infected with H9N2 avian influenza virus (AIV) compared with mock infection. The differentially expressed genes (DEGs), and the data of GO enrichment analysis and KEGG pathway analysis for DEGs were reported here. In addition, some of these DEGs associated with innate immune response and antigen presentation were also verified by qPCR. The replication of H9N2 AIV in DCs, and the viability kinetic of DCs during H9N2 AIV infection, and the primers for qPCR were also reported in this data article. The data presented here was used on the research article entitled “Transcriptomic profile of chicken bone marrow-derive dendritic cells in response to H9N2 avianinfluenza A virus”.

Specifications table

**Table:** 

Subject	Immunology and Microbiology
Specific subject area	The virus infection influence on the gene expression of immune calls.
Type of data	Table
	Figure
How data were acquired	RNA-seq via Illumina HiSeq XTen (Illumina, USA), Real-time PCR via Thermal Cycler DICE Real-Time System Lite TP700 (Takara, Japan)
Data format	Raw data, analyzed
Parameters for data collection	Mock and H9N2 AIV infected bone marrow derived DCs from chickens
Description of data collection	Changes in the gene expression of chicken DCs for H9N2 virus infection
Data source location	Key Laboratory of Veterinary Biological Engineering and Technology, Ministry of Agriculture, Nanjing, Jiangsu, China
Data accessibility	Raw data of RNA Seq analysis were deposited to NCBI, and the GEO accession numbers is GSE117163.
Related research article	Liu Q et al., 2020, Transcriptomic profile of chicken bone marrow-derive dendritic cells in response to H9N2 avian influenza A virus, Vet Immunol Immunopathol, 220: 109,992 [1].

**Value of the Data**

• The first global gene expression analysis of chicken DCs infected with H9N2 AIV.
• These data will help to understand the host immune response to H9N2 infection in chickens.
• Expression analysis data in chicken DCs may be further used for comparative analysis with expression assays in other poultry.

## 1 Data description

**Fig. 1:**
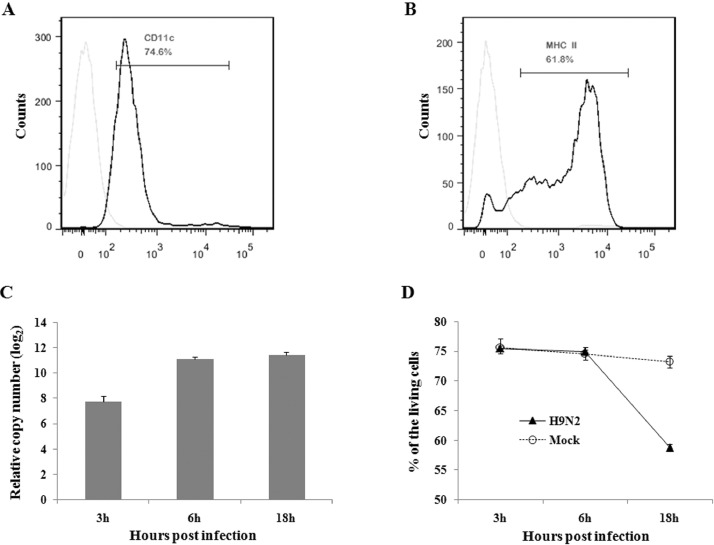
Phenotype identification and H9N2 AIV infection of BM-DCs. The surface molecule MHC Class II (A) and CD11c (B) on the BM-DCs were determined by flow cytometric analysis using mouse anti-human CD11c antibody (eBioscience, USA) or mouse anti-chicken MHCII antibody (Abcam, USA). (C) BM-DCs were infected with the H9N2 AIV, and the total RNA was isolated from H9N2 AIV infected BM-DCs for analysis of the viral M1-specific RNAs by SYBR Green real-time PCR. The expression levels of viral M1 gene at 3, 6, 18 h post infection are presented as the relative gene expression in relation to that at 0.5 h post infection, which represent the increases of viral RNA levels during the time course of infection. The results are presented as means from triplicate measurements with standard deviations. (D) H9N2 AIV and mock infected BM-DCs were collected at 3, 6, 18 h and stained with Annexin V for flow cytometric analysis. Data on percentage of living cells is presented as mean values from triplicate measurements with standard deviations.

**Fig. 2:**
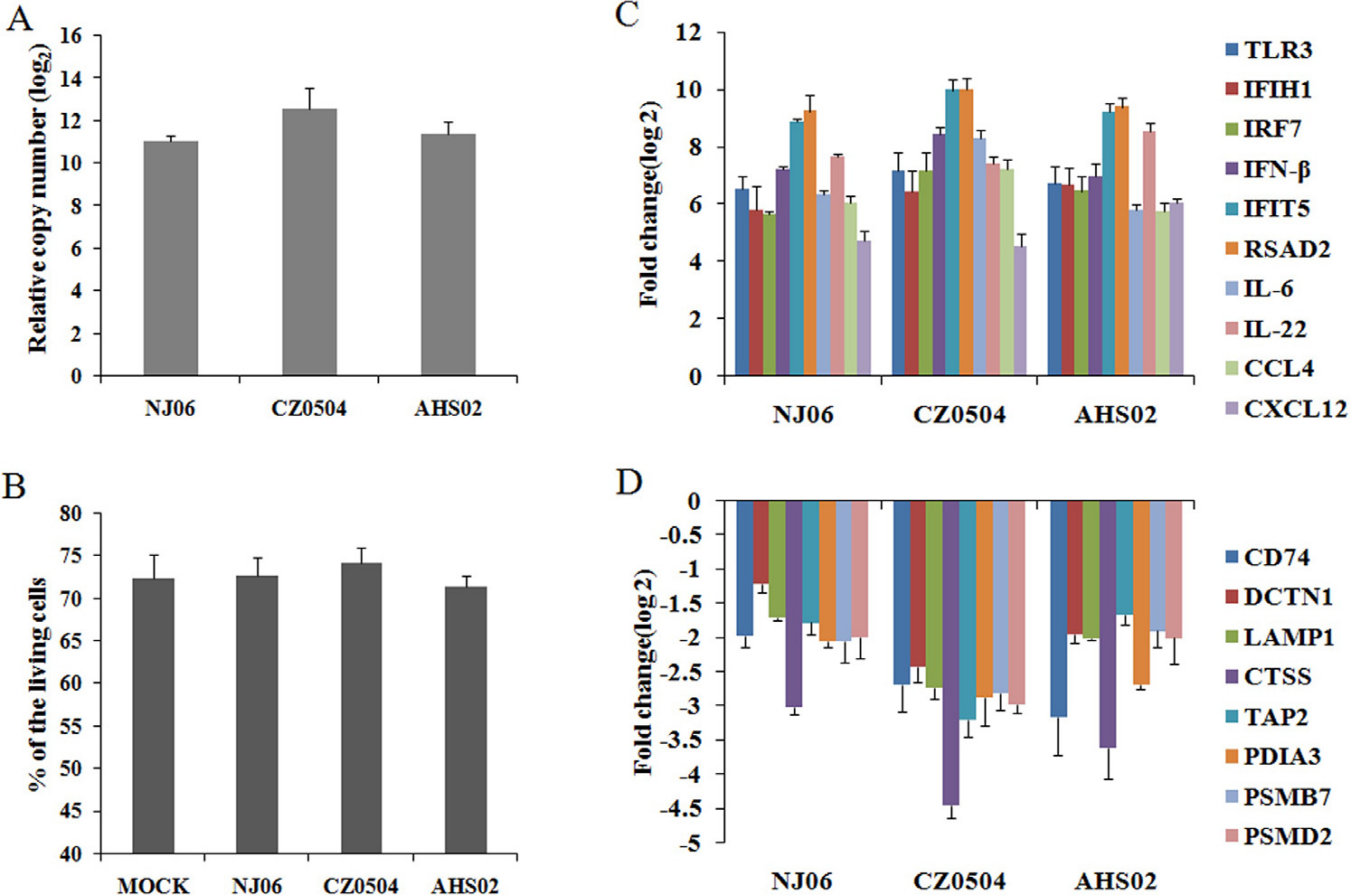
BM-DCs were infected with A/duck/Nanjing/06/2003(NJ06), A/chicken/Changzhou/0504/2017(CZ0504), and A/chicken/Anhui/S02/2013(AHS02) H9N2 AIV strains and were collected at 6 h post infection. (A) The total RNA was isolated from BM-DCs for analysis of the viral M1-specific RNAs by SYBR Green real-time PCR and the expression levels of viral M1 gene are presented as the relative gene expression in relation to that at 0.5 h post infection. The results are presented as means from triplicate measurements with standard deviations. (B) H9N2 AIV and mock infected BM-DCs were collected and stained with Annexin V for flow cytometric analysis. Data on percentage of living cells is presented as mean values from triplicate measurements with standard deviations. (C and D) The DEGs involved in host innate immune responses and antigen presentation were selected to be confirmed by qPCR. The histograms indicate the qPCR data which are expressed as the means standard deviations (SD) for triplicate infections.

Here we report the global gene expression analysis data of chicken DCs infected with H9N2 AIV compared with mock infection. The sequence database was deposited to NCBI, and the GEO accession numbers is GSE117163. The data show that 4151 genes were significantly up-regulated, and 2138 genes were significantly down-regulated following H9N2 AIV infection (Supplementary Table 1). GO enrichment analysis of these differentially expressed genes (DEGs) showed that a total of 130 and 120 GO terms were significantly enriched for the up- and down-regulated DEGs respectively, in three main GO categories: cellular components, molecular functions, and biological processes (Table 1, Supplementary Tables 2–4). Pathway analysis of the up-regulated and down-regulated DEGs was also performed on the KEGG database (Table 2, Supplementary Table 5). In addition, the phenotype identification of DCs, and the viability kinetic of DCs during H9N2 AIV infection, and the replication of H9N2 AIV in DCs and some of these DEGs were also determined by flow cytometric analysis and qPCR (Figs. 1 and 2), and the primers for qPCR were listed in Table 3.

**Table 1:** GO terms significantly enriched by up- or down-regulated DEGs for biological process.

GO ID	GO Term	DEGs style	-log_10_FDR
GO:0,006,811	ion transport	Up	9.526894014
GO:0,055,085	transmembrane transport	Up	8.465671499
GO:0,007,186	G-protein coupled receptor signaling pathway	Up	8.129413176
GO:0,007,268	synaptic transmission	Up	7.380059989
GO:0,007,267	cell-cell signaling	Up	7.084386694
GO:0,007,275	multicellular organismal development	Up	6.287296285
GO:0,006,936	muscle contraction	Up	5.973701573
GO:0,034,765	regulation of ion transmembrane transport	Up	5.783910618
GO:0,007,601	visual perception	Up	5.708395794
GO:0,042,391	regulation of membrane potential	Up	5.147163012
GO:0,007,155	cell adhesion	Up	5.131140366
GO:0,030,198	extracellular matrix organization	Up	4.205396928
GO:0,071,805	potassium ion transmembrane transport	Up	4.195542284
GO:0,007,165	signal transduction	Up	3.76675983
GO:0,006,813	potassium ion transport	Up	3.708036028
GO:0,034,220	ion transmembrane transport	Up	3.605281638
GO:0,042,472	inner ear morphogenesis	Up	3.271156529
GO:0,010,951	negative regulation of endopeptidase activity	Up	3.191259258
GO:0,007,602	phototransduction	Up	2.683349047
GO:0,007,218	neuropeptide signaling pathway	Up	2.657366515
GO:0,007,154	cell communication	Up	2.621742773
GO:0,006,814	sodium ion transport	Up	2.552679391
GO:0,030,818	negative regulation of cAMP biosynthetic process	Up	2.305595239
GO:0,007,605	sensory perception of sound	Up	2.287929054
GO:0,007,166	cell surface receptor signaling pathway	Up	2.176002285
GO:0,050,953	sensory perception of light stimulus	Up	1.856059697
GO:0,006,812	cation transport	Up	1.836750255
GO:0,051,216	cartilage development	Up	1.752329879
GO:0,030,049	muscle filament sliding	Up	1.750389712
GO:0,019,229	regulation of vasoconstriction	Up	1.750389712
GO:0,001,974	blood vessel remodeling	Up	1.711364263
GO:0,007,187	G-protein coupled receptor signaling pathway, coupled to cyclic nucleotide second messenger	Up	1.698685935
GO:0,070,588	calcium ion transmembrane transport	Up	1.570097419
GO:0,009,607	response to biotic stimulus	Up	1.561075333
GO:0,009,612	response to mechanical stimulus	Up	1.539448937
GO:0,008,272	sulfate transport	Up	1.527518462
GO:1,902,358	sulfate transmembrane transport	Up	1.458586361
GO:0,030,154	cell differentiation	Up	1.436427558
GO:0,019,532	oxalate transport	Up	1.433603081
GO:0,007,204	positive regulation of cytosolic calcium ion concentration	Up	1.419354807
GO:0,015,701	bicarbonate transport	Up	1.404425818
GO:0,035,725	sodium ion transmembrane transport	Up	1.386341211
GO:0,070,098	chemokine-mediated signaling pathway	Up	1.359987939
GO:0,009,653	anatomical structure morphogenesis	Up	1.344405489
GO:0,002,027	regulation of heart rate	Up	1.339913059
GO:0,050,896	response to stimulus	Up	1.323489649
GO:0,031,018	endocrine pancreas development	Up	1.306589409
GO:0,008,152	metabolic process	Down	13.17523377
GO:0,000,278	mitotic cell cycle	Down	11.09472543
GO:0,044,281	small molecule metabolic process	Down	6.260417925
GO:0,055,114	oxidation–reduction process	Down	4.690525623
GO:0,042,590	antigen processing and presentation of exogenous peptide antigen via MHC class I	Down	3.526556283
GO:0,002,474	antigen processing and presentation of peptide antigen via MHC class I	Down	3.506546589
GO:0,031,145	anaphase-promoting complex-dependent proteasomal ubiquitin-dependent protein catabolic process	Down	3.506546589
GO:0,005,975	carbohydrate metabolic process	Down	3.26355729
GO:0,006,260	DNA replication	Down	3.152057253
GO:0,000,082	G1/S transition of mitotic cell cycle	Down	3.114065771
GO:0,044,255	cellular lipid metabolic process	Down	3.064571406
GO:0,051,439	regulation of ubiquitin-protein ligase activity involved in mitotic cell cycle	Down	3.064571406
GO:0,006,457	protein folding	Down	2.822434026
GO:0,006,418	tRNA aminoacylation for protein translation	Down	2.776822875
GO:0,007,094	mitotic spindle assembly checkpoint	Down	2.776822875
GO:0,007,067	mitotic nuclear division	Down	2.768732327
GO:0,002,479	antigen processing and presentation of exogenous peptide antigen via MHC class I, TAP-dependent	Down	2.69855525
GO:0,006,200	ATP catabolic process	Down	2.602969703
GO:0,007,049	cell cycle	Down	2.524100463
GO:0,034,976	response to endoplasmic reticulum stress	Down	2.444228149
GO:0,006,271	DNA strand elongation involved in DNA replication	Down	2.444228149
GO:0,007,076	mitotic chromosome condensation	Down	2.341198832
GO:0,043,277	apoptotic cell clearance	Down	2.341198832
GO:0,015,031	protein transport	Down	2.315189616
GO:0,006,270	DNA replication initiation	Down	2.273274234
GO:0,006,629	lipid metabolic process	Down	2.26057764
GO:0,051,437	positive regulation of ubiquitin-protein ligase activity involved in mitotic cell cycle	Down	2.14441609
GO:0,051,436	negative regulation of ubiquitin-protein ligase activity involved in mitotic cell cycle	Down	2.14441609
GO:0,030,968	endoplasmic reticulum unfolded protein response	Down	2.130960444
GO:0,030,261	chromosome condensation	Down	2.094490573
GO:0,044,267	cellular protein metabolic process	Down	2.083055589
GO:0,043,687	post-translational protein modification	Down	2.013949561
GO:0,006,099	tricarboxylic acid cycle	Down	1.953559525
GO:0,006,508	proteolysis	Down	1.866941451
GO:0,006,509	membrane protein ectodomain proteolysis	Down	1.781469722
GO:0,051,301	cell division	Down	1.751025857
GO:0,006,635	fatty acid beta-oxidation	Down	1.657618606
GO:0,032,201	telomere maintenance via semi-conservative replication	Down	1.51911888
GO:0,033,540	fatty acid beta-oxidation using acyl-CoA oxidase	Down	1.51911888
GO:0,019,885	antigen processing and presentation of endogenous peptide antigen via MHC class I	Down	1.51911888
GO:0,006,665	sphingolipid metabolic process	Down	1.444993539
GO:0,090,382	phagosome maturation	Down	1.376321186
GO:0,006,687	glycosphingolipid metabolic process	Down	1.36012919
GO:0,051,701	interaction with host	Down	1.36012919
GO:0,030,433	ER-associated ubiquitin-dependent protein catabolic process	Down	1.313495682

**Table 2:** KEGG pathways significantly enriched by up- or down-regulated DEGs.

Pathway ID	Pathway Term	DEGs style	(-log_10_FDR)
PATH:04,080	Neuroactive ligand-receptor interaction	Up	9.228962756
PATH:04,060	Cytokine-cytokine receptor interaction	Up	7.94406601
PATH:04,512	ECM-receptor interaction	Up	3.380229513
PATH:04,610	Complement and coagulation cascades	Up	2.860948
PATH:04,744	Phototransduction	Up	2.636047378
PATH:04,974	Protein digestion and absorption	Up	2.323949981
PATH:04,950	Maturity onset diabetes of the young	Up	2.323949981
PATH:04,975	Fat digestion and absorption	Up	1.709952575
PATH:00,830	Retinol metabolism	Up	1.709952575
PATH:04,350	TGF-beta signaling pathway	Up	1.532818581
PATH:04,151	PI3K-Akt signaling pathway	Up	1.458023559
PATH:04,020	Calcium signaling pathway	Up	1.458023559
PATH:04,724	Glutamatergic synapse	Up	1.458023559
PATH:05,217	Basal cell carcinoma	Up	1.458023559
PATH:04,978	Mineral absorption	Up	1.458023559
PATH:05,033	Nicotine addiction	Up	1.458023559
PATH:00,591	Linoleic acid metabolism	Up	1.326413177
PATH:04,142	Lysosome	Down	9.91673
PATH:01,100	Metabolic pathways	Down	6.428568
PATH:01,200	Carbon metabolism	Down	5.71436
PATH:04,145	Phagosome	Down	5.411231
PATH:04,141	Protein processing in endoplasmic reticulum	Down	4.305033
PATH:00,970	Aminoacyl-tRNA biosynthesis	Down	4.021603
PATH:03,030	DNA replication	Down	3.303353
PATH:00,020	Citrate cycle (TCA cycle)	Down	2.92431
PATH:00,640	Propanoate metabolism	Down	2.92431
PATH:04,110	Cell cycle	Down	2.37307
PATH:03,430	Mismatch repair	Down	2.300895
PATH:03,410	Base excision repair	Down	2.228318
PATH:00,531	Glycosaminoglycan degradation	Down	1.872496
PATH:00,071	Fatty acid degradation	Down	1.833489
PATH:01,230	Biosynthesis of amino acids	Down	1.782571
PATH:00,030	Pentose phosphate pathway	Down	1.497989
PATH:00,860	Porphyrin and chlorophyll metabolism	Down	1.397797
PATH:01,212	Fatty acid metabolism	Down	1.325871
PATH:01,210	2-Oxocarboxylic acid metabolism	Down	1.325871

**Table 3:** Primers used for qPCR.

Primer name	Sequene(5′−3′)	GenBank accession no.
CCL4-F	CCTCATCCAGAGGCACTACA	NM_204,720.1
CCL4-R	GCTTGACGCTCTGCAGGTA	
CCL20-F	TCTGCCTCGGAAGGTCATTA	NM_204,438.2
CCL20-R	AGGATTTACGCAGGCTTTCA	
CCL28-F	GGCCTTTAACTGTTGCACGA	XM_015272682
CCL28-R	ATCCGTGGGCTTACACAGAA	
CXCL12-F	CTGGCAGTCATCTCCCTGTC	NM_204,510.1
CXCL12-R	AATCTGAAGCGAGCAGTTGG	
CXCLi1-F	CGGACCTCACTGCAAGAATG	NM_205,018.1
CXCLi1-R	GCCTTGTCCAGAATTGCCTT	
CXCLi2-F	CTGCGGTGCCAGTGCATTAG	NM_205,498.1
CXCLi2-R	AGCACACCTCTCTTCCATCC	
IL-1β-F	TGCTTCGTGCTGGAGTCAC	HQ329098.1
IL-1β-R	GGCATCTGCCCAGTTCCA	
IL-6-F	GCTACAGCACAAAGCACCTG	HM179640.1
IL-6-R	GACTTCAGATTGGCGAGGAG	
IL-12A-F	TGGCCGCTGCAAACG	AY262751.1
IL-12A-R	ACCTCTTCAAGGGTGCACTCA	
IL-12B-F	AGATGCTGGCAACTACACCTG	NM_213,571.1
IL-12B-R	CATTTGCCCATTGGAGTCTAC	
IL-17F-F	CTAGCTGTGCTGCAGTGTTC	JQ776598.1
IL-17F-R	AAGCTTCTCAGAGCCACCAT	
IL-22-F	CAGACTCATCGGTCAGCAAA	AJ617782.1
IL-22-R	GGTACCTCTCCTTGGCCTCT	
IRF7-F	ACATGTTCATGCTGCTGGAG	KP096419.1
IRF7-R	GAGTGTTTTCCAAGGCCAAA	
IFIH1-F	ATTCTGGGACTTACAGCCTCAC	NM001193638.1
IFIH1-R	ACCTGATTCTTCAGTTGGGATG	
IFN-β-F	CTTGCCCACAACAAGACGTG	AY974089.1
IFN-β-R	GTGTTTTGGAGTGTGTGGGC	
IFN-κ-F	GAGAAATTGGAGGCGTGCAT	KR817821.1
IFN-κ-R	CATTTCTGCACGGCTGATCT	
OASL-F	GGTCTACGTGAAGCTGTTGG	NM_205,041.1
OASL-R	GTCTTTCAGCTTAGCAGGGC	
RSAD2-F	TGGTCAAGGAAGGAAGAAC	NM_001318443.1
RSAD2-R	TGATTAGGCACTGGAACAC	
IFIT5-F	TGCTTCACCAGCTAGGACTCTGC	KT180229.1
IFIT5-R	TGGCTTTTGCTCTGTCACCACTTTG	
IFN-γ-F	TGAGCCAGATTGTTTCGATG	DQ906156.1
IFN-γ-R	CTTGGCCAGGTCCATGATA	
TLR3-F	GCAACACTTCATTGAATAGCCTTGAT	MF576162.1
TLR3-R	TTCAGTATAAGGCCAAACAGATTTCC	
DDX60-F	ACCGTGCCTCAGTGTTTAGA	XM_004940918.3
DDX60-R	TCCCAAACCTCTGCTCCAAT	
DHX58-F	AGCCCACGAAGCAGTACGA	MF563593.1
DHX58-R	CGGCAACTCGGGCATCT	
IFITM3-F	ATCGCAAAGTCCTGGGTG	NM_001350061.1
IFITM3-R	TGCTGCTGGTGGTTGAAGA	
PDIA6-F	GTTGGTCGTGGTTCTACAGC	XM_419,952.5
PDIA6-R	CCGGAAGCTCACCATCTTTG	
PDIA3-F	TGCTACAGCCAATGATGTGC	NM_204,110.3
PDIA3-R	TCCCGCTTCAAGTAGCTGAT	
TAP1-F	TCGTCACCTTCCTCCTCTACCA	JF794482.1
TAP1-R	CCCGGTCCAGGAACTCAAA	
TAP2-F	CGTCCCACCGTCCTTATCCT	JF794489.1
TAP2-R	CTTCTCCAGCATCCGTGGTT	
TAPBP-F	ACGTCTACAGCTGCGTTGTCA	NM_001034816.3
TAPBP-R	AGGACAAAGGCCACCAAGAA	
PSMB7-F	ACAGCTGCAGACACTGAGAT	NM_204,397.1
PSMB7-R	AGCACCAATGTAGCCCTGAT	
PSMC1-F	GAGGGAGATCCAGCGTACAA	NM_204,958.1
PSMC1-R	GTCGATACGTCCTGGCCTAA	
PSMC2-F	GCTGGTGCAGAAGTATGTGG	NM_001006225.1
PSMC2-R	ACCATCATCAAAGCGAGCAC	
PSMC3-F	CAGGAGGAGGATGGAGCAAA	NM_001031190.1
PSMC3-R	ACCCGATAACAGGCAGGAAA	
PSMD2-F	ACCTGCTCATGGAGATCGAG	NM_001012934.1
PSMD2-R	AACGGTTGAACTTGCGGAAA	
PSMD3-F	TCTCCTTCTGCCTCGACATC	NM_001031362.1
PSMD3-R	TCATCGTCCTCTGCCATCTC	
TPP2-F	GACTCTGCGAGGAACACAAC	XM_004938558.2
TPP2-R	CGTTAGCTTTGAGCCCTGAC	
LAMP1-F	CAACGTGACTTTGGAAGCCT	NM_205,283.2
LAMP1-R	ACCTGGCTAGTAGCGTGTTT	
M6PR-F	ATAGTGGGAGCAAAGGGCAT	XM_416,477.6
M6PR-R	ACATCGGTAGCAAGTGGTCA	
TCIRG1-F	CCTCTGGATGGTGCTGTTTG	NM_204,722.1
TCIRG1-R	GAAGCCGGTGTAGATGGAGA	
CTSB-F	CCAAGCTCCCTGAAAGGGTA	NM_205,371.2
CTSB-R	GAGCCCTGGTCTCTGATCTC	
CTSK-F	GCAACGAGAAGGCTCTGAAG	NM_204,971.2
CTSK-R	GGTTGATGTTCTCCGGGTTG	
CTSS-F	CTGCCCTCAAAGATGCTGTC	NM_001031345.1
CTSS-R	TTTCACCAGCCAGAAGTCCT	
CD74-F	CAATCCCAGCGGAGAAAGTG	NM_001001613.1
CD74-R	TAGTCACCGTTCTCATCGCA	
DCTN1-F	AAGCTGGAGACGCTGAAGAT	NM_001031367.1
DCTN1-R	CCTGCATCTTGCTCTTCCAC	
IFI30-F	CCGCCACCAAGAATCTGAAG	XM_418,246.5
IFI30-R	TTCCCATTGATGACGACCCA	
LGMN-F	GCTGCAGAGATGAAAGCACA	XM_015287754.1
LGMN-R	GCAGTCGTAGTTGCTGATGG	
RACGAP1-F	CCAGGCAGTATGTTGAAGGC	XM_004949732.3
RACGAP1-R	TGAGAGTCGTCTTTGCCACT	
UNC93B1-F	TCTACACGCCTGTCCTCATC	XM_015286086.1
UNC93B1-R	TGAAGTAGCGCTCCCAGTAG	
H9N2 AIV M1-F	CGAATGGGAACGGTAACCAC	KX349958.1
H9N2 AIV M1-R	GCCATCTGTCTGTGAGACCT	
Actinb-F	GTGATGGACTCTGGTGATGG	NM_205,518.1
Actinb-R	TGGTGAAGCTGTAGCCTCTC	

## 2 Experimental design, materials, and methods

### 2.1 Cell culture and virus infection

The bone marrow (BM) monocytes were collected from femurs of four 4-week-old specific pathogen-free (SPF) white leghorn chickens, and were cultured for dendritic cells (BM-DCs) as previously described, with some modifications [2]. Briefly, BM cells were cultured in 6-well plates at a concentration of 5 × 10^6^/ml in RPMI-1640 (Wisent) complete medium containing 5% FBS (Wisent), 100 U/ml penicillin and 100 µg/ml streptomycin for 6 h at 41 °C in 5% CO_2_, and then non-adherent cells were removed by replacing with fresh complete medium containing 50 ng/ml chicken GM-CSF (Abcam, USA), and 10 ng/ml IL-4 (Kingfisher, USA). Half of the medium was replaced with fresh complete medium containing GM-CSF and IL-4 at day 2, 4 and 6. At day 7, the surface markers of BM-DCs were analyzed by flow cytometry with antibodies as previous experiments [3], and then BM-DCs were used for the infection of H9N2 AIV.

Three H9N2 subtype avian influenza viruses, A/duck/Nanjing/06/2003(NJ06), A/chicken/Changzhou/0504/2017(CZ0504), and A/chicken/Anhui/S02/2013(AHS02), were propagated in SPF white leghorn chicken eggs respectively, and the allantoic fluid was concentrated via sucrose gradient ultracentrifugation and resuspended in RPMI. The allantoic fluid from mock infected eggs was processed in the same manner and used for mock infection. Viral titers were measured by calculating the 50% tissue culture infectious dose (TCID_50_) in MDCK cells. Unless otherwise stated in the text, H9N2 AIV refers to NJ06. BM-DCs (2 × 10^6^/ml) were infected with H9N2 AIV (10^6^ TCID_50_/0.1 ml) and then were collected for RNA sequencing at 6 h post infection. Three independent biological replicates of the cell culture and virus infection experiments were performed for the RNA sequencing analysis.

### 2.2 RNA sequencing

Total RNA was extracted from BM-DCs using TRIzol reagent (Invitrogen). The integrity and concentration of the extracted RNA were assessed by Agilent 2200 Bioanalyzer (Agilent Technologies, USA). RNA samples with RNA Integrity Number ≥ 7 were used for library construction. The libraries were prepared using the TruSeq RNA Sample Preparation Kit (Illumina, USA) according to the manufacturer's protocol, and then sequenced on the Illumina HiSeq XTen (Illumina, USA). The construction and sequencing of libraries were performed by Shanghai Bioinformatics (Shanghai, China), and the GEO accession numbers for the RNA-seq data is GSE117163.

### 2.3 Data analysis

The raw reads were filtered by removing the adaptor sequences and low-quality reads containing more than 5% ambiguous bases (noted as N) or more than 20% of bases with qualities of <20 to obtain clean reads. Thereafter, the clean reads were mapped to the Chicken genome (Version: Gallus_gallus-5.0 NCBI), using HISAT2 with default parameter. The gene expression data were generated and normalized by fragments per kilobase of transcript per million uniquely mapped reads (FPKM) [4]. Differentially expressed genes (DEGs) analysis was performed using DEGSeq algorithm, and DEGs with a p-value <0.05, a FDR <0.05 and a fold change >2 were selected for GO and KEGG pathway enrichment analyses, respectively. The GO and KEGG pathways were considered significantly enriched when FDR < 0.05.

### 2.4 Quantitative real-time PCR

The DEGs recognized by RNA-seq was verified by Quantitative real-time PCR (qPCR). Total RNA was isolated from a replica RNA sequencing infection experiment using TRIzol reagent (Life Technologies) and treated with DNase I (Fermentas, Glen Burnie, MD, USA). One microgram of total RNA per sample was reverse transcribed into cDNA using a PrimeScript RT Reagent Kit (Takara). The qPCR was performed using Talent qPCR PreMix SYBR Green (Tiangen, China) on a Real-Time System Lite TP700 (Takara, Japan). The product specificity of qPCR was verified by one cycle for melting curve analysis. The expression of each cytokine gene relative to that of the β-actin was calculated using the 2^−△△CT^ method. All primers for these target genes are listed in Table 3.

## Appendix Supplementary materials

Image, application 1
